# Characteristics and Treatment of Anaphylaxis in Children Visiting a Pediatric Emergency Department in Korea

**DOI:** 10.1155/2020/2014104

**Published:** 2020-02-27

**Authors:** Won Seok Lee, Jaewoo An, Young-Ho Jung, Hye Mi Jee, Kyu-Young Chae, Young A. Park, Man Yong Han, Kyung Suk Lee

**Affiliations:** ^1^Department of Pediatrics, CHA Ilsan Medical Center, CHA University, Goyang, Republic of Korea; ^2^Pediatric Emergency Department, CHA Ilsan Medical Center, CHA University, Goyang, Republic of Korea; ^3^Department of Pediatrics, CHA Bundang Medical Center, CHA University, Seongnam, Republic of Korea; ^4^Pediatric Emergency Center, CHA Bundang Medical Center, CHA University, Seongnam, Republic of Korea; ^5^Seongbok Seoul Asan Pediatric Clinic, Gyeonggi-do, Republic of Korea; ^6^Department of Pediatrics, Hanyang University Guri Hospital, Hanyang University, Gyeonggi-do, Republic of Korea

## Abstract

Anaphylaxis is a serious life-threatening allergic disease in children. This study is aimed at determining the characteristics of pediatric patients who experienced anaphylaxis along with treatments administered in order to determine the usefulness of tryptase level assessment as a marker of anaphylaxis in Korean children. A total of 107 patients who were diagnosed with anaphylaxis in a single pediatric emergency center over a 3-year period were included in the study. Patient clinical characteristics, symptoms, signs, allergy history, trigger factors, treatments, and laboratory findings, including serum tryptase levels, were included in the analysis. Food allergies (39.3%) were the most commonly reported patient allergic history, and 58 patients (54.2%) were triggered by food. Among this group, nuts and milk exposure were the most common, affecting 15 patients (25.9%). History of anaphylaxis and asthma were more common in severe anaphylaxis compared to mild or moderate anaphylaxis cases. Epinephrine intramuscular injection was administrated to 76 patients (71.0%), and a self-injectable epinephrine was prescribed to 18 patients (16.8%). The median tryptase level was 4.80 ng/mL (range: 2.70–10.40) which was lower than the 11.4 ng/mL value commonly documented for standard evaluation in adults with anaphylaxis. The most common cause of pediatric anaphylaxis was food including nuts and milk. The rate of epinephrine injection was relatively high in our pediatric emergency department. The median tryptase level associated with anaphylaxis reactions in children was lower than 11.4 ng/mL. Further studies are needed to help improve diagnostic times and treatment accuracy in pediatric patients who develop anaphylaxis.

## 1. Introduction

Anaphylaxis is a rapidly progressing systemic allergic disease characterized by severe immediate hypersensitivity reaction [1]. Without rapid and appropriate treatment, severe symptoms affecting various organ systems can occur within a short period of time [2]. Hospitalization rates due to anaphylaxis across all age groups increased from 21.0 to 25.1 cases per million between 1999 and 2009, and hospitalizations due to food-induced anaphylaxis in patients less than 18 years of age doubled in the United States during this same time period [3, 4]. The incidence of anaphylaxis in children is higher [1] compared to adults, and incidence increases as age decreases [5].

Diagnosis of anaphylaxis is mainly based on symptoms, history, physical examination, and blood tests including tryptase level analysis [6, 7]. Furthermore, drugs and insect stings are the most common anaphylaxis triggers in adults [8]. The epidemiology of childhood anaphylaxis triggers has been shown to be different from that of adults with the most common cause of childhood anaphylaxis being food [5].

The most effective treatment of anaphylaxis is intramuscular administration of epinephrine [7]. However, the rate of epinephrine administration for cases of anaphylaxis is relatively low resulting from a lack of standardized protocols, low awareness of guidelines, and misplaced concerns regarding the safety of epinephrine [9].

Anaphylaxis is known to be mediated predominantly by secretion of tryptase and cytokines from mast cells [10]. Observation of tryptase levels during the anaphylactic reaction and postreaction periods may be helpful in diagnosis [11]. Several studies in adults have shown that tryptase levels had significant diagnostic value for determining cases of anaphylaxis, and elevated tryptase levels were associated with severe anaphylaxis [12, 13]. A pediatric study reported that tryptase levels were significantly related with severe anaphylaxis or anaphylaxis due to milk allergies; however, tryptase levels in pediatric anaphylaxis were generally lower than the cutoffs commonly used in standard evaluations (≥11.4 ng/mL) [14]. In Korea, a limited number of studies have examined pediatric anaphylaxis, and investigations regarding the association between pediatric anaphylaxis and tryptase levels have not been reported. The purpose of this study was to determine the characteristics and treatments administered in cases of pediatric anaphylaxis, in order to assess the usefulness of tryptase level assessments as a diagnostic marker of anaphylaxis in Korean children.

## 2. Material and Methods

### 2.1. Patients

This study evaluated the medical records of 80,981 patients who visited a single pediatric emergency center between January 1, 2015, and December 31, 2017, for study inclusion. Our pediatric emergency department is an urgent medical care center geared specifically to the needs of children under 15 years of age in the local community. Of these, 146 patients had anaphylactic-related International Classification of Disease (ICD) codes (ICD-10 codes: T780, T782, and T886). From this initially recruited study population, 107 patients who met the diagnostic criteria of anaphylaxis based on the National Institutes of Health symposium [7] definition were included in the final study population and underwent retrospective medical record review by pediatricians. This study was approved by the Institutional Review Board (IRB) of CHA University Bundang CHA Hospital (IRB number 2018-04-023).

### 2.2. Severity of Anaphylaxis

Severity of anaphylaxis (mild, moderate, and severe) was classified according to a 3-point modified grading system published by Brown [15]. Mild anaphylaxis was defined as the presence of skin and subcutaneous symptoms (urticaria, redness, and angioedema) as well as oral pruritus, nausea (i.e., gastrointestinal involvement), or nasal congestion, sneezing, rhinorrhea, or throat tightness (i.e., respiratory involvement) [15]. Moderate anaphylaxis was defined as the presence of any of the previous symptoms as well as abdominal cramping, recurrent vomiting or diarrhea, dyspnea, stridor, coughing, wheezing, or light headedness [15]. Severe anaphylaxis was defined as the presence of cyanosis, hypoxia (oxygen saturation < 92%), respiratory arrest, hypotension, dysrhythmia, confusion, or change in mental status [15].

### 2.3. Anaphylactic Patient Characteristics and Hematologic Profiles

A total of 107 anaphylactic patients were retrospectively analyzed, and information regarding age, sex, time to hospital visit following symptom onset, allergy history, family allergy history, trigger factors, emergency signs and symptoms, treatment, severity, and laboratory findings including white blood cell counts, eosinophil counts, total IgE, tryptase (Thermo Fisher Scientific, Uppsala, Sweden), and ImmunoCAP for allergen-specific IgE (Thermo Fisher Scientific, Uppsala, Sweden) levels were collected. Among the patients with anaphylaxis, tryptase tests were performed in 25 patients, and two samples were excluded from analysis since they were measured more than 3 hours following symptom onset [12, 13].

Differences in clinical manifestation and laboratory tests between the tryptase tested and untested groups were analyzed. The tryptase-tested group was divided into patients with a value at or above 11.4 ng/mL and patients with a value of less than 11.4 ng/mL. Subsequently, differences in age, sex, allergy history, family history of allergy, clinical signs and symptoms, laboratory findings, and treatments were also analyzed. Correlation analyses were performed to examine the association of tryptase levels with ImmunoCAP.

### 2.4. Statistical Analysis

Results were statistically analyzed using the Mann-Whitney *U* test for continuous variables and chi-square test for nominal variables. The Spearman correlation method was used for correlation analysis. All data were analyzed using SPSS version 25.0 (IBM, New York, NY) software.

## 3. Results

### 3.1. Clinical Characteristics of Study Subjects and Trigger Factors of Anaphylaxis

**Figure 1:**
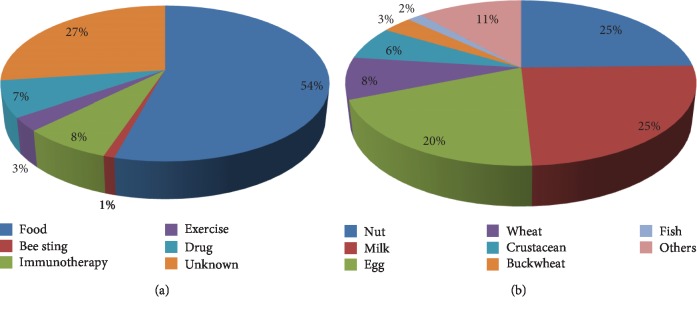
Trigger factors and food triggers of patients who visited the pediatric emergency department with anaphylaxis. (a) Trigger factors and (b) food triggers.

Patient clinical characteristics and observations are summarized in Table 1. Among patients who experienced anaphylaxis, 63 (58.9%) were male and the median age was 4.0 years (interquartile range: 1.0–8.0 years). Because the ages of patients were not normally distributed, we expressed ages in interquartile ranges. Common signs and symptoms in patients included skin rash in 92 (86.0%), dyspnea in 73 (68.2%), and facial edema in 68 (63.6%). Furthermore, 70 (66.7%) patients had a history of allergies, and 42 (39.3%) patients had a history of food allergies. With regard to additional allergy history, 34 (31.8%) had atopic dermatitis, 27 (25.2%) had allergic rhinitis, 19 (17.8%) had asthma, and anaphylaxis was observed in 6 (5.6%) patients. Thirty-six patients (33.6%) had a documented family history of allergy. Allergy trigger factors included food in 58 (54.2%), unknown cause in 29 (27.1%), and drugs and immunotherapy in 8 (7.5%) patients. For patients with food-triggered anaphylaxis, common triggers included nuts in 15 patients (25.9%), milk in 15 patients (25.9%), and eggs in 12 patients (20.7%) (Figure 1).

Based on the anaphylaxis severity classification, the number of patients in the mild, moderate, and severe anaphylaxis groups were 3 (2.8%), 77 (72.0%), and 27 (25.2%), respectively. We compared the mild and moderate anaphylaxis groups with the severe anaphylaxis group and found statistically significant differences between the two groups with regard to history of anaphylaxis and asthma (Table 1).

### 3.2. Treatment of Anaphylactic Patients

As shown in Table 2, intramuscular epinephrine injections were administered to 76 patients (71.0%), systemic steroid was used in 93 patients (86.9%), and antihistamines were administered in 89 patients (83.2%). Self-injectable epinephrine was prescribed for 18 patients (16.8%) in outpatient follow-up after discharge.

### 3.3. Laboratory Findings

Analysis of laboratory findings showed no significant differences in white blood cell counts, eosinophil percentages, C-reactive protein, total IgE, and ImmunoCAP levels between the anaphylaxis groups. ImmunoCAP showed sensitization in 61 cases (91.0%) of which 29 (55.8%) were sensitized to inhalation allergens and 42 patients (80.8%) were sensitized to food allergens. The median value of tryptase for the 23 patients that received the tryptase test was 4.8 ng/mL (range: 2.70–10.40 ng/mL) (Table 3).

### 3.4. Comparison between Tryptase Level Groups and Correlation Analysis between Tryptase and Other Laboratory Findings

There were no significant differences with regard to age, sex, allergy history, or clinical symptoms when comparing between the high tryptase level (≥11.4 ng/mL) and low tryptase level (<11.4 ng/mL) groups. However, there was a significant positive correlation between elevated tryptase levels and buckwheat-specific antigen in the ImmunoCAP tests (rho = .890, *P* = 0.018, data not shown).

## 4. Discussion

This study evaluated the clinical characteristics, emergency treatments, and tryptase levels in anaphylactic patients who presented to a pediatric emergency department in Korea. To our knowledge, this is the first study to evaluate the treatment of pediatric anaphylactic patients who presented to the pediatric emergency department in order to assess tryptase level changes in Korean children. Our study found that most of the tryptase levels in pediatric patients with anaphylaxis were less than half of the commonly used reference value (≥11.4 ng/mL) [16, 17]. It was also noted that elevated tryptase levels were not always associated with a more severe clinical manifestation when compared to lower tryptase levels.

The accurate diagnosis of anaphylaxis is not trivial since it is dependent on symptoms and suggestive history after exposure to potential triggers. Anaphylaxis symptoms are known to vary and most commonly include cutaneous symptoms (urticaria, angioedema) along with dyspnea, wheezing, syncope, and vomiting [18]. Our study found that cutaneous symptoms were the most common symptom, while dyspnea, wheezing, and vomiting were relatively common; however, syncope and hypoxia occurred with relatively low frequency. Emesis is known to be more common in children [12], and our study also found that vomiting was reported in a large proportion of patients.

It is known that adverse drug reactions and insect stings are the most common anaphylaxis triggers in adults [8] while the most common cause of childhood anaphylaxis is food [5, 8]. According to one study, nuts, including peanuts and cashews, were the most common food triggers in pediatric anaphylaxis cases [5]. In our study, the most common food triggers in Korean children were nuts, milk, and eggs, which was similar to what has been previously documented [5, 19].

Since the diagnosis of anaphylaxis can be challenging, the accurate and timely treatment of anaphylaxis cases can present some obstacles. Worldwide allergy society guidelines state that intramuscular epinephrine delivered to the anterolateral thigh is the first-line treatment for anaphylaxis [7]. The alpha_1_-adrenergic effects of epinephrine treat shock, decrease airway edema, and promote mast cell stabilization, which subsequently decreases the release of histamine. In addition to this, the beta_2_-adrenergic effects of epinephrine result in bronchodilation [20]. In a US study examining pediatric anaphylaxis cases, epinephrine was administered in 44% of emergency department cases [9]. In another recent US study, the rate of epinephrine use in the emergency department for pediatric anaphylaxis was found to be 47% [13]. Russell et al. proposed that the reasons for the discordance between guidelines and practice were a lack of standardized protocols, low awareness of guidelines, or mistaken concerns regarding the safety of epinephrine [21].

Our study showed that the rate of epinephrine administration was 71% and was found to be higher than in previous studies [9, 13]. Educated medical staff and prompt decisions of treatment of anaphylactic patients by pediatric or emergency specialists were the reasons for the high injection rate in our center. One of the prior mentioned US studies reported that self-injectable epinephrine administration rates before hospital arrival (at home or in the ambulance) was 15% [13]. According to another recent US study, the rate of patients who were previously prescribed with epinephrine was 35.4% and the rate of prescribed epinephrine administration prior to emergency department presentation was 68.1% [22]. On the other hand, our study found that none of the self-injectable epinephrine syringes were administered by patients or caregivers before the arrival to the hospital. In Korea, this may be a result of a lack of education regarding treatment of anaphylaxis or may be a result of ambulances not having access to epinephrine. In patients treated at our emergency department for anaphylaxis, self-injectable epinephrine was prescribed in 16.8% of cases after discharge. Although a self-injectable epinephrine is prescribed, it is not easy to administer properly when anaphylaxis occurs outside of the hospital. Patients often do not carry the self-injectable epinephrine [23]. Consequently, self-injectable epinephrine should be more widely prescribed, and it is also necessary to educate the public regarding their proper use.

For treatments besides epinephrine in this study, systemic steroids were used in 86.9%, antihistamine in 83.2%, and respiratory nebulized therapy in 20.6% of patients. The 2010 National Institute of Allergy, Immunology, and Infectious Diseases (NIAID) guidelines reported a lack of evidence for using glucocorticoids and antihistamines as routine medications for anaphylaxis [24]. However, in the clinical setting, glucocorticoids and antihistamines are frequently administered [25]. The effects of glucocorticoids are well-established for asthma or airway edema [26]. A recent study showed a reduced risk of prolonged hospitalization in pediatric anaphylaxis cases who received glucocorticoids [27]. While histamine_1_ (H_1_) blockers such as diphenhydramine can reduce urticaria, they do not alter the underlying pathophysiology of anaphylaxis [25]. Therefore, the administration of antihistamines as adjuvant treatment has been shown to be reasonable in providing improved comfort, especially for patients with urticaria or angioedema [25]. In addition, there are no previous studies examining the efficacy of histamine_2_ (H_2_) blockers for anaphylaxis [28], and the effect of such use is not clear [29].

During anaphylaxis, mast cells and basophils release mediators including histamine, tryptase, chymase, cytokines, and chemokines [10]. Histamine measurements must be performed within 15 to 60 minutes after anaphylaxis onset [1]. However, tryptase levels can be measured between 15 minutes and 3 hours after symptom onset resulting in a more sensitive diagnosis of anaphylaxis [1]. Serum tryptase levels within the first 3 hours of symptom onset may serve as a selective marker for anaphylaxis [6]. However, tryptase tests usually take an extended period of time for result confirmation, presenting a limitation for use in the emergency department setting. Regardless, tryptase tests could be useful for confirmation of anaphylaxis as well as during patient follow-up after admission or discharge.

There are currently no internationally set criteria for interpretation of serum tryptase tests for acute anaphylactic reactions, although ≥11.4 ng/mL is the most frequently used global cutoff value [16, 17]. Several studies have been conducted regarding associations of serum tryptase levels and anaphylaxis, particularly with regard to the severity of adult anaphylaxis [10, 30]. To our knowledge, there is only one Canadian study that described tryptase levels in pediatric anaphylaxis cases, which showed that anaphylaxis severity and milk-induced anaphylaxis were associated with tryptase level changes [14]. However, our study did not find a statistically significant association between the characteristics of anaphylaxis and ≥11.4 ng/mL tryptase levels.

Basophils are mainly involved in food-induced anaphylaxis compared to mast cells, and since food is the main cause of anaphylaxis in children, tryptase levels may often be normal in pediatric anaphylaxis cases [11]. Our study found a positive correlation between elevation of tryptase levels and ImmunoCAP sensitization to buckwheat-specific antigen. Further studies are needed to examine how specific food allergens affect tryptase levels and the relationship between food allergen and tryptase in anaphylaxis.

There are several limitations to our study. First, we did not compare our anaphylaxis group tryptase level measurements to a normal control group, and the number of enrolled patients in this study was relatively small. Second, we could not establish a causal relationship since this study was conducted retrospectively. However, this study was the first of its kind to investigate treatment in pediatric anaphylaxis cases and to uniquely describe the measured tryptase levels in anaphylaxis cases of Korean children.

## 5. Conclusions

This study found that the most common cause of pediatric anaphylaxis was food including nuts and milk. The rate of epinephrine intramuscular injection was found to be relatively high in this study, and tryptase levels in children with anaphylaxis was lower than those reported in adult studies. There were statistically significant differences between mild to moderate and severe anaphylaxis groups with regard to syncope, hypotension, cyanosis, and history of anaphylaxis and asthma. Tryptase levels were not significantly associated with patient allergic history or clinical symptoms. Accurate and prompt administration of epinephrine for treatment of anaphylaxis in pediatric emergency departments is needed in addition to increased provision of prescriptions and administration of self-injectable epinephrine. Future studies enrolling pediatric patients who have experienced anaphylaxis are needed to help improve accuracy of diagnosis and treatment. These studies should include an assessment of the utility of tryptase levels in the diagnosis of anaphylaxis in pediatric cases.

## Figures and Tables

**Table 1:** Clinical characteristics of anaphylaxis and comparison of mild to moderate with severe anaphylaxis in patients who visited the pediatric emergency department (*n* = 107).

	Total (%)	Mild to moderate (*n* = 80)	Severe (*n* = 27)	*P* value
Sex (male) (%)	63 (58.9)	49 (61.2)	14 (51.9)	0.391
Age (median, years)^†^	4.0 (1.0–8.0)	4.0 (1.0–8.0)	4.0 (1.0–9.0)	0.089
History of allergic disease (%)	70 (66.7)	53 (66.2)	17 (63.0)	0.756
Anaphylaxis	6 (5.6)	2 (2.5)	4 (14.8)	0.034^∗^
Asthma	19 (17.8)	18 (22.5)	1 (3.7)	0.038^∗^
Urticaria	2 (2.0)	1 (1.2)	1 (3.7)	0.443
Drug allergy	5 (4.7)	3 (3.8)	2 (7.4)	0.598
Food allergy	42 (39.3)	31 (38.8)	11 (40.7)	1.000
Allergic rhinitis	27 (25.2)	20 (25.0)	7 (25.9)	1.000
Atopic dermatitis	34 (31.8)	26 (32.5)	8 (29.6)	1.000
Oral allergy syndrome	1 (0.9)	1 (1.2)	0 (0.0)	1.000
Familial history of allergic disease (%)	36 (33.6)	27 (33.8)	9 (33.3)	0.968
Symptoms and signs				
Headache	2 (1.9)	1 (1.2)	1 (3.7)	0.443
Dizziness	1 (0.9)	0 (0.0)	1 (3.7)	0.252
Dyspnea	73 (68.2)	58 (72.5)	15 (55.6)	0.150
Wheeze	34 (31.8)	26 (32.5)	8 (29.6)	1.000
Throat tightness	15 (14.0)	14 (17.5)	1 (3.7)	0.108
Rash	92 (86.0)	68 (85.0)	24 (88.9)	0.756
Facial edema	68 (63.6)	52 (65.0)	16 (59.3)	0.647
Abdominal pain	9 (8.4)	6 (7.5)	3 (11.1)	0.689
Nausea	3 (2.8)	3 (3.8)	0 (0.0)	0.570
Vomiting	28 (26.2)	20 (25.0)	8 (29.6)	0.622

∗ indicates a *P* value of <0.05, and † indicates interquartile range.

**Table 2:** Treatment of anaphylactic patients who visited the pediatric emergency department (*n* = 107).

	Patient number (%)
Epinephrine (%)	76 (71.0)
Steroid (%)	93 (86.9)
Antihistamine (%)	89 (83.2)
Nebulizer (%)	22 (20.6)
Self-injectable epinephrine prescription (%)	18 (16.8)

**Table 3:** Laboratory findings including ImmunoCAP tests of anaphylaxis patients who visited the pediatric emergency department.

	Total	Mild to moderate (*n* = 80)	Severe (*n* = 27)	*P* value
Tryptase (ng/mL) (*n* = 23)	4.80 (2.70–10.40)			
WBC (×10^3^/*μ*L)	13,169 (9,410–17,580)	12,060 (8,510–16,023)	11,055 (8,233–14,755)	0.455
Eosinophil (×10^3^/*μ*L)	1.0 (0.5–3.0)	1.0 (0.7–3.1)	0.9 (0.5–2.0)	0.150
Total IgE (UI/mL)	132.9 (44.5–379.2)	145.2 (47.9–336.8)	120.8 (46.6–565.05)	0.131
CRP (mg/dL)	0.04 (0.00–0.20)	0.04 (0.00–0.18)	0.09 (0.03–0.23)	0.635
ImmunoCAP (positive, %)	61/67 (91.0)	46/49 (93.9)	15/18 (83.3)	0.180
Inhalant allergen (positive, %)	29/52 (55.8)	22/39 (56.4)	7/13 (53.8)	0.872
Food allergen (positive, %)	42/52 (80.8)	30/36 (83.3)	12/16 (75.0)	0.482

∗ indicates a *P* value of <0.05. WBC: white blood cell; CRP: C-reactive protein.

## Data Availability

All data used to support the findings of this study are included within the article.
